# Genome-Wide Comparative Analysis of HIF Binding Sites in *Cyprinus Carpio* for *In Silico* Identification of Functional Hypoxia Response Elements

**DOI:** 10.3389/fgene.2019.00659

**Published:** 2019-07-16

**Authors:** Iliyas Rashid, Ajey Kumar Pathak, Ravindra Kumar, Prachi Srivastava, Mahender Singh, S Murali, Basdeo Kushwaha

**Affiliations:** ^1^Molecular Biology and Biotechnology Division, ICAR-National Bureau of Fish Genetic Resources, Lucknow, India; ^2^AMITY Institute of Biotechnology, AMITY University Uttar Pradesh, Lucknow, India

**Keywords:** genome, gene promoter, gene enhancer, hypoxia-inducible factor, hypoxia response element, hypoxia ancillary sequence, HREExplorer

## Abstract

*Cyprinus carpio* is world’s most widely distributed freshwater species highly used in aquaculture. It is a hypoxia-tolerant species as it lives in oxygen-deficient environment for a long period. The tolerance potential of an animal against hypoxia relates it to induced gene expression, where a hypoxia-inducible factor (HIF) binds to a transcriptionally active site, hypoxia response element (HRE), a 5-base short motif that lies within the promoter/enhancer region of a certain gene, for inducing gene expression and preventing/minimizing hypoxia effects. HRE is functionally active when it contains another motif, the hypoxia ancillary sequence (HAS), which is typically adjacent to downstream of HRE within 7- to 15-nt space. Here, an attempt was made for mining HRE and identifying functional HIF binding sites (HBS) in a genome-wide analysis of *C. carpio*. For this, gene information along with the 5,000-nt upstream (−4,900 to +100) sequences of 31,466 protein coding genes was downloaded from “Gene” and “RefSeq” databases. Analysis was performed after filtration of the impracticable genes. A total of 116,148 HRE consensus sequences were mined from 29,545 genes in different promoter regions. HRE with HAS consensus motifs were found in the promoter region of 9,589 genes. Further, the already reported genes for hypoxia response in humans and zebrafish were reanalyzed for detecting HRE sites in their promoters and used for comparative analysis with gene promoters of *C. carpio* for providing support to identify functional HBS in the gene promoter of *C. carpio*. An interactive user interface HREExplorer was developed for presenting the results on the World Wide Web and visualizing possible HBS in protein coding genes in *C. carpio* and displaying the comparative results along with the reported hypoxia-responsive genes of zebrafish and reported hypoxia-inducible genes in humans. In this study, a set of Perl program was written for the compilation and analysis of information that might be used for a similar study in other species. This novel work may provide a workbench for analyzing the promoter regions of hypoxia-responsive genes.

## Introduction

*Cyprinus carpio*, commonly known as common carp, is a worldwide distributed freshwater carp fish of Family Cyprinidae, having vulnerable conservation status, and is a widely cultivated aquaculture species contributing about 10% to the total freshwater aquaculture production. It is a hypoxia-tolerant species that lives for a prolonged period in oxygen-depleted environment and reported for different physiologic and metabolic adaptations under hypoxia, including renal (Kakuta et al., 1992), cardiorespiratory (Stecyk and Farrell, 2002), and cellular and molecular (Tasaki et al., 2017) responses.

Hypoxia is a condition of oxygen deficiency in the body tissues, which affects proper metabolic activity, alters the expression of several genes, and causes various abnormalities in different animals, ranging from invertebrates to mammals (Guillemin and Krasnow, 1997). Environmental hypoxia is common in fishes due to the increase in water temperature, presence of organic pollutants, aquatic flora that consume ambient oxygen, depth of water column, water flow, and other physical factors that create heterogeneous aquatic environment with imbalanced oxygen concentration in aquatic ecosystems (Nilsson and Ostlund-Nilsson, 2004). Fishes encounter hypoxia more frequently than the air-breathing animal and can adopt tolerability potential through natural selection to preserve oxygen contents and cellular energy for hypoxia survival. Their adaptation is associated with behavioral responses, inducing higher hematocrit and altering/reducing metabolic activity to preserve more oxygen for survival during hypoxia and maintenance of oxygen homeostasis (Perry et al., 2009; Vornanen et al., 2009; Richards, 2011). Evolutionary adaptation is not uniform in fishes and the physiology and the metabolic processes are altered significantly under hypoxic conditions in several species (Thetmeyer et al., 1999; Padmavathy et al., 2009). Hypoxia-sensitive fish attempt to meet high metabolic demands with insufficient ATP during hypoxia without any further change in the behavioral and physiologic responses that can consequently lead to even mortality.

Organs, tissues, and cells of the tolerant species may cope up with the low ambient oxygen in the medium to maintain homeostasis for adaptation to the conditions. Several oxygen-dependent genes produce essential proteins with induced expression mechanism during hypoxia in many fishes for maintaining metabolic and physiologic activities (Semenza, 2009). The upstream region of a gene creates a basal transcriptional machinery of the genes in eukaryotes, which includes promoter. A promoter region is started immediately to the upstream from the transcription start site (TSS) categorized as core, proximal, and distal regions and the diverse gene uses a distinct promoter type (Riethoven, 2010). The core promoter elements, located near the TSS, are the most important regions to initiate gene expression (Smale and Kadonaga, 2003; Carninci et al., 2006). The proximal promoter region in a gene, located next to the core promoter toward upstream and typically composed of CpG islands and several motifs, each with a distinct pattern for binding with a specific transcription factor (TF), is involved in the regulation process for gene expression (Butler and Kadonaga, 2002, Sarda et al., 2017). The distal promoter may be located many kilobases far from the TSS of a gene and is composed of several regulatory elements, including enhancers (up-regulation), silencers (down-regulation), and insulators (protective regulation), which possess protein binding sites or TF binding site (TFB) for TF (Roeder, 1991). Earlier studies revealed that a nuclear transcriptional factor or hypoxia-inducible factor (HIF) binds at TFB in the gene promoter for transcriptional activation of the human EPO gene (Semenza and Wang, 1992). Rainbow trout is the leading fish in which the expression and function of HIF-1α are studied (Soitamo et al., 2001).

HIF recognizes hypoxia response elements (HREs), a TFB within the promoters of a large number of genes, and binds to it after hypoxic induction and enhances the expression of the gene (Wenger et al., 2005). HREs are normally distributed in the genome (Dengler et al., 2014) and their frequent occurrences were reported in the enhancer/promoter and 3′ untranslated regions of a gene (Fordel et al., 2004). Functional HREs have been reported in several mammalian genes (Kimura et al., 2001), whereas few studies have proposed functional HREs in fishes (Rees et al., 2001, Rees et al., 2009). The lists of confirmed HIF target genes is expanding gradually. The functionally active HREs have been observed in the promoter region of more than 100 mammalian genes involved in different biological processes (Manalo et al., 2005). Frequently distributed HREs are functionally activated when its downstream region contains an adjacent hypoxia ancillary sequence (HAS) in the space of 7 to 15 nt (Kajimura et al., 2006; Liu et al., 1995).

HRE (5′-RCGTG-3′) and HAS [5′-CA(G|C)(A|G)(T|G|C)-3′] are 5-nt motifs whose regular expression pattern from the 5′ end are described as follows: in the first and second places, two bases, C and A, are conserved; the third place is G or C; the fourth place is any purine base; and fifth place is one base from T or G or C. The consensus HRE elements with a preceding C on 5′, like 5′-CRCGTG-3′, form the enhancer box (E-box) binding sites of TFs of other basic helix-loop-helix (bHLH) protein families, and HIF rarely recognizes to bind this site similar to HRE. The HAS consensus patterns have been reported in several hypoxia-inducible genes (HIGs) in mammals and IGFBP1 and EPO genes in fish (Kimura et al., 2001; Kajimura et al., 2006; Kulkarni et al., 2010). Numerous HIGs, existing with hypoxia-binding sites, were identified in humans and zebrafish using genome-wide analysis approaches (Ortiz-Barahona et al., 2010; Greenald et al., 2015). This information can be used to identify HIG in other related species using computational approaches (Benita et al., 2009).

In the present study, a genome-wide analysis of *C. carpio* was carried out for i) mining of HIF binding site (HBS) in the 5 kb upstream sequences of protein coding genes reported in *C. carpio*, ii) predicting the functional site in the promoter regions of protein coding genes, iii) identifying HRE along with HAS elements, iv) obtaining HRE frequency with the E-box promoter binding site, and v) performing comparative analysis for validating the findings using information on identified HIGs in zebrafish, a model fish belonging to the same Cyprinidae family and having well-annotated genome assembly with 52,610 protein coding genes (Xu et al., 2014), and in humans. An information browser called HREExplorer was developed using Linux, Apache, MySQL, PHP, and Perl (LAMPP) to visualize the information on each HRE located in the promoter region of the protein coding genes of *C. carpio*, and a hyperlink was given to the GenBank database for obtaining the sequences of a particular region. The frequency of HRE distribution and location was identified in the core, proximal, and distal regions of the upstream sequences. This study may provide insights into the molecular events that link reduced oxygenation to HIF activation and may serve as a platform for analyzing genes in hypoxia susceptible fishes of aquaculture importance. Additionally, it will be helpful in evaluating HIGs in phylogenetically relative species.

## Materials and Methods

### Data Collection

**Figure 1:**
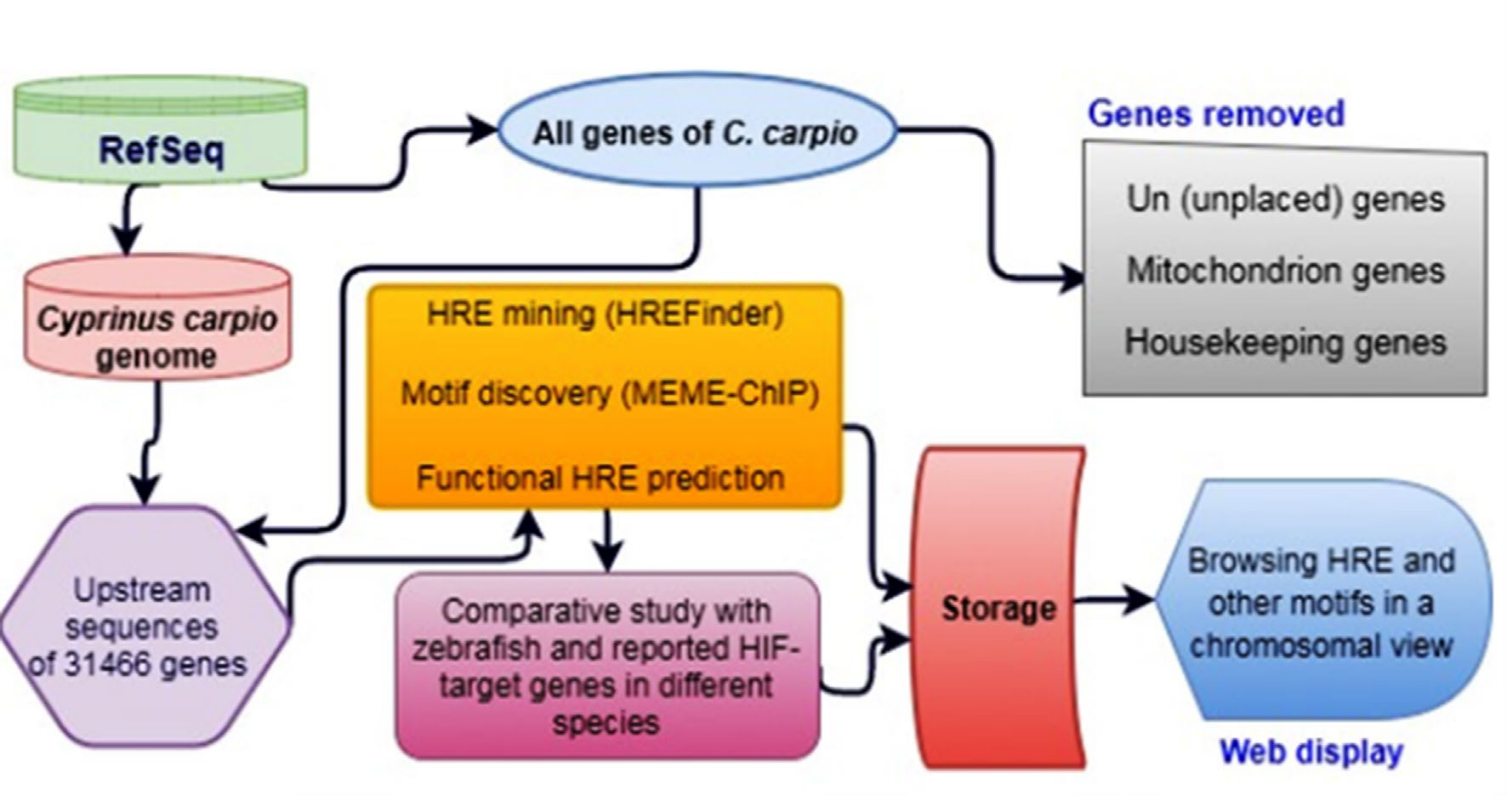
Data flow diagram of genome-wide hypoxia response element (HRE) analysis in *C. carpio*.

Gene database (Brown et al., 2015) and genomes of *C. carpio* and zebrafish were downloaded from GenBank and FASTA file formats from the latest release of the National Center for Biotechnology Institute (NCBI) FTP site (NCBI Resource Coordinators, 2017) using “wget” utility under Linux environment. A total of 69,131 summarized gene information, including gene and placed coordinates with plus/minus orientation, of *C. carpio* (NCBI Taxon ID: 7962) were extracted from the downloaded gene database using an in-house Perl script. The methodology of genome-wide HRE mining and analysis in *C. carpio* is illustrated in a data flow diagram presented in **Figure 1**. HREs were mined only in the 5 kb upstream of gene localized strands and the complementary strands were not used for the analysis.

### Gene Filtration

A large number of *C. carpio* genes were extracted from the gene database named here as “extracted genes (EG).” The EG without defining a location on a chromosome (genomic sequence) was represented as unplaced in the gene database. The upstream sequences of such genes could not be parsed for this study and thus were discarded from the EG. Mitochondrial genes were also discarded as they were beyond the scope of this study. The housekeeping genes (HKGs) were also filtered out from the EG of *C. carpio* using the downloaded reference of HKGs in humans (Eisenberg and Levanon, 2013). In this process, the reference human HKGs contained 3,804 gene symbols (https://m.tau.ac.il/∼elieis/HKG/) that were matched with the gene symbol of *C. carpio* using an in-house developed Perl program. It was found that 890 gene symbols of HKGs were exactly the same in the EG that were removed. Further, the remaining HKGs did not match with the gene symbol for most of the genes because many genes are defined with LOC+gene ID in *C. carpio*. To remove the remaining HKGs from the EG, the HKG symbols of humans were first compared to the gene symbols of zebrafish annotated genome to obtain the mRNA IDs of those genes in zebrafish. The mRNA IDs of zebrafish HKGs were used to download mRNA sequence from the Batch Entrez. The formatdb of Blast package (Altschul et al., 1990; Mount, 2007) was used to create Blast-compatible database “HKdb” for the downloaded mRNA sequences of zebrafish. Further, all the mRNA sequences from the EG of *C. carpio* were mapped onto HKdb using the BlastN program and significantly aligned gene sequences were removed from the EG list of *C. carpio*.

### Dataset Preparation

After filtration of unplaced, mitochondrial, and HKGs, 31,466 genes remained in the EG that included 15,635 plus and 15,831 minus genomic orientations along with the localization coordinates on the chromosome. Further, gene orientation information was used to parse upstream sequences from the respective chromosome (genomic sequences) using the methodology applied for parsing the upstream of all genes localized either in forward or reverse strand in genomic DNA (Rashid et al., 2017a, Rashid et al., 2017b). The upstream parsing program included reverse complement mechanism in methodology for parsing of genes localized on the minus strand of the DNA helix. This has prepared a dataset of all uniformly parsed sequences in the 5′ to 3′ direction. In this process, a collection of 5,000 nt (−4,900 to +100) upstream sequence of each gene was obtained.

### Comparative Data Analysis

A list of 737 genes differentially expressed (442 down-regulated and 295 up-regulated) under hypoxia exposure was reported in Supplementary file 1 in the genome-wide mapping of HIF1α binding sites in zebrafish by Greenald et al. (2015). The list of reported genes is available with the manuscript in the form of a data sheet composed of different fields, including UniGene ID as sequence identifiers. However, the information on the sequences and the HRE positions in the upstream regions of these reported genes were lacking; thus, the available information on reported genes delimited to use the data for comparative analysis with HRE contained promoters of *C. carpio*. The given UniGene ID in the above study was only a gene identifier for HIG in zebrafish. To overcome this, an extensive meta-analyses was performed for the reported genes of zebrafish, which included i) identifying the sequence accession number and gene ID from UniGene ID, ii) downloading and parsing of upstream sequences, iii) mining of HRE element and their localized orientation in different promoter regions, and iv) computing the total number of HRE with HAS in those upstream sequences. Thus, the gene IDs of all 737 genes were obtained from the UniGene dataset of zebrafish using the reference of UniGene ID given in the data sheet. Further, gene location on the chromosome, genomic accessions, and the gene orientation along with the other gene details were obtained from the gene database for those reported genes. Finally, 5 kb upstream sequences for all the 737 genes were parsed from the corresponding genomic sequences using information of gene location and orientation. HRE and HRE with HAS were mined and their genomic localization was computed along with the orientation in the promoter region of those genes. After this reanalysis of the reported hypoxia response genes (RHRG) of zebrafish, a dataset was prepared to perform a comparative analysis of the predicted HBS in the promoter region of *C. carpio*.

Similarly, a list of 30 HIG genes in humans was collected from a literature survey for performing comparative analysis of promoter regions with genes of *C. carpio*. The gene information and genomic sequences were downloaded from the NCBI. Upstream sequences were parsed using the gene localization and orientation information. HREFinder was used to mine HRE consensus within upstream sequences.

### Orthologous Gene Identification

Finding homologous genes in *C. carpio* against the RHRG of zebrafish was a big challenge for performing comparative analysis between the promoters of the same genes in both species. The methodology for matching the exact gene symbol is not an appropriate practice for the identification of homologous genes, as there are so many orthologous genes reported for both species but with different gene symbols, e.g. the gene symbol for vascular endothelial growth factor is “vegfab” in zebrafish and “LOC109065938” in *C. carpio*; similarly, the gene symbol for the gene acot11 acyl-CoA thioesterase 11 is “acot11a” in zebrafish but “acot11” in *C. carpio*. Further, a close examination of the data obtained from zebrafish used in the comparative study revealed numerous genes without published gene symbols. Apart from authentic gene symbols, there are also many sets of alphanumeric temporary gene symbols started with LOC, Si, zgc, and znrf followed by numbers. The exact symbol-matching algorithm failed here for recognizing those varying patterns of gene symbol. A homology-based comparison (Pearson, 2013) was performed between the mRNA sequences of RHRG of zebrafish and all the mRNA sequences of *C. carpio* genes taken in the study with 80% identity. In this homology search, an identity threshold cutoff was considered because mRNA sequences on 80% identity with conserved functional sites generally share the same function.

### Tools and Techniques

Several in-house scripts were written in Perl for the preparation, manipulation, and compilation of datasets for analysis (**Table 1**). HREFinder (http://mail.nbfgr.res.in/HRGFish/download/), a tool for discovering regular expression-based consensus motifs, was used here for the mining consensus pattern of HRE in the upstream region of the genes. An extensive analysis of the mined HRE was done in different ways. A data file was generated using an in-house Perl program “MemeAnalysisDataset.pl” for the functional analysis of the HRE motifs through MEME-ChIP, a position weight matrix-based tool for motif identification (Machanick and Bailey, 2011) having the ability of short TFB identification with high accuracy rate (Tanaka et al., 2014). This in-house program used the position of the HRE in the upstream and parsed 40-nt sequences, which included 10 nt upstream and 30 nt downstream from the HRE end position. A list of sequences with uniform length containing HRE was generated as a query for MEME-ChIP. The VENNY tool (Oliveros, 2007–2015) was employed for depicting the HRE mining results in a Venn diagram. LAMPP package was used for storing information about genes, chromosomes, upstream, and HRE related data using the InnoDB storage engine in the back-end and the user interfaces were designed for viewing and browsing information in the front-end.

**Table 1:** List of in-house Perl programs designed and implemented for genome-wide hypoxia response element (HRE) mining in *C. carpio* and comparative study with human and zebrafish.

S.N.	Program	Description
1.	HKgeneMapper.pl	Mapping program that uses a list of human HKGs to match identical gene symbols with *C. carpio* and zebrafish. The results of this program produces the matched gene symbol list of *C. carpio* and zebrafish (HKG).
2.	SpmRNAIDsCollect.pl	Collects mRNA IDs of listed genes.
3.	BlastParse.pl	Performs alignment of all mRNA of *C. carpio* with “DrHKdb,” a Blast dataset of mRNA of HKGs of zebrafish. It generates a list of HKG ID of *C. carpio* on significant Blast hits.
4.	GenomicAccList.pl	Generates lists of “GeneIDs” and genomic accession IDs.
5.	ParseGenomicFileGBnFASTA.pl	Splits the downloaded genomic files into individual genomic sequences in.fasta and.gb format
6.	UpstreamParse.pl	Parses upstream sequences of genes using reverse complement method for minus strand.
7.	BaseCount.pl	Counts the nucleotide bases and computes CG percentage of one or multiple.fasta format sequences given in a single file.
8.	HREFinder.pl	Computes the motifs HRE and HRE with HAS along with their localized information in different promoter regions in a set of promoter (upstream) sequences.
9.	PositionEvaluate.pl	Uses output file of HREFinder.pl to evaluate localization coordinates of gene, upstream, 30 nt HRE sub-string, HRE motif, HAS motif, and E-box motif on chromosome.
10.	GFFFileGeneUpstrm.pl	Generates.gff file of gene and upstream in the genome.
11.	GFFFileHREandHAS.pl	Generates.gff file for HRE motifs and HRE with HAS in the upstream of genes.
12.	MotifClust.pl	Generates separate input files for Venny to cluster results in a Venn diagram.
13.	MemeAnalysisDataset.pl	Generates a homologous dataset with 100 nt of either side of HRE from different upstream sequences for MEME analysis.
14.	DatabaseCreation.pl	Creates a different table of “carphre” database for storing the record.
15.	UniGene2geneid.pl	Uses UniGene dataset of zebrafish to extract gene ID and record.
16.	GeneID2mRNAID.pl	Generates a list of mRNA IDs, including all variants of gene.
17.	ParsemRNAFileGBnFASTA.pl	Splits each mRNA file into separate GB and.fasta file.
18.	mRNAID2GeneID.pl	Uses mRNA accession from alignment file and generates their gene list.
19.	HsCc_ComparativeAnalysis.pl	Performs comparative analysis between mRNA of HIG of human and mRNA of all protein coding genes of *C. carpio*.
20.	GeneratesHumanHIGinfotable.pl	Generates HIG information along with computed information on upstream and HRE site.

## Results

### HRE Mining in the Promoter Region

**Figure 2:**
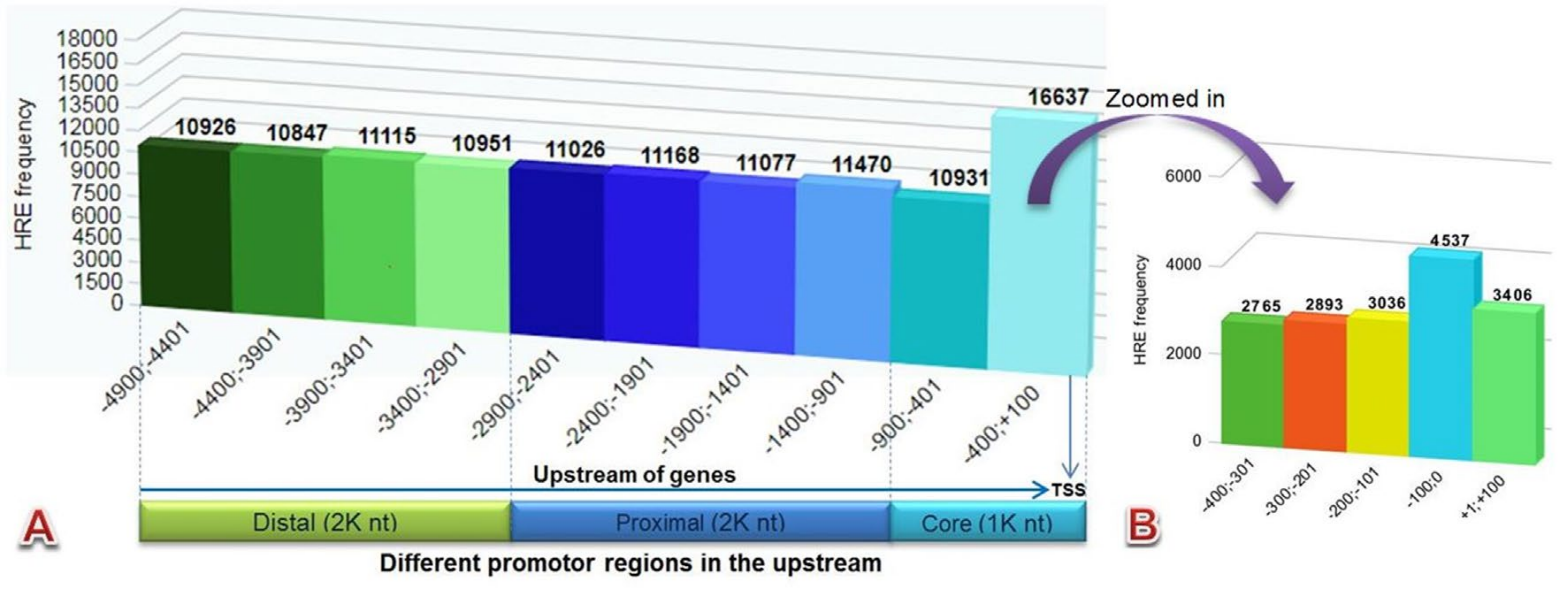
Bar diagram showing HRE distribution pattern of **(A)** all canonical HREs in different promoter regions **(B)** zoomed in –400;+100 region.

The HRE motif (5′-RCGTG-3′) pattern in 29,545 (94%) genes was found using the HREFinder tool that was distributed in different promoters in 5 kb (−4,900 to +100) upstream sequences of 31,466 EGs of *C. carpio*. A total of 116,148 HRE consensus were examined, as there were several genes that had more than one HRE in their upstream. The distribution frequency of the HREs in the upstream regions was found almost uniform, except in the core promoters, particularly near the TSS, where it was higher. The identified motif in different promoter regions included 43% distal (2,000 nt), 38% proximal (2,000 nt), and 19% core (1,000 nt), respectively, as presented in **Figure 2A**. Higher numbers of HRE distribution were found near the TSS in the region −400;+100 and **Figure 2B** presents the zoomed-in image of the HRE distribution in this region. A total of 9,589 genes were found that contained HREs with HAS consensus motif located within 15 bases of their downstream.

### Meta-Analyses for Genome-Wide HRE

**Figure 3:**
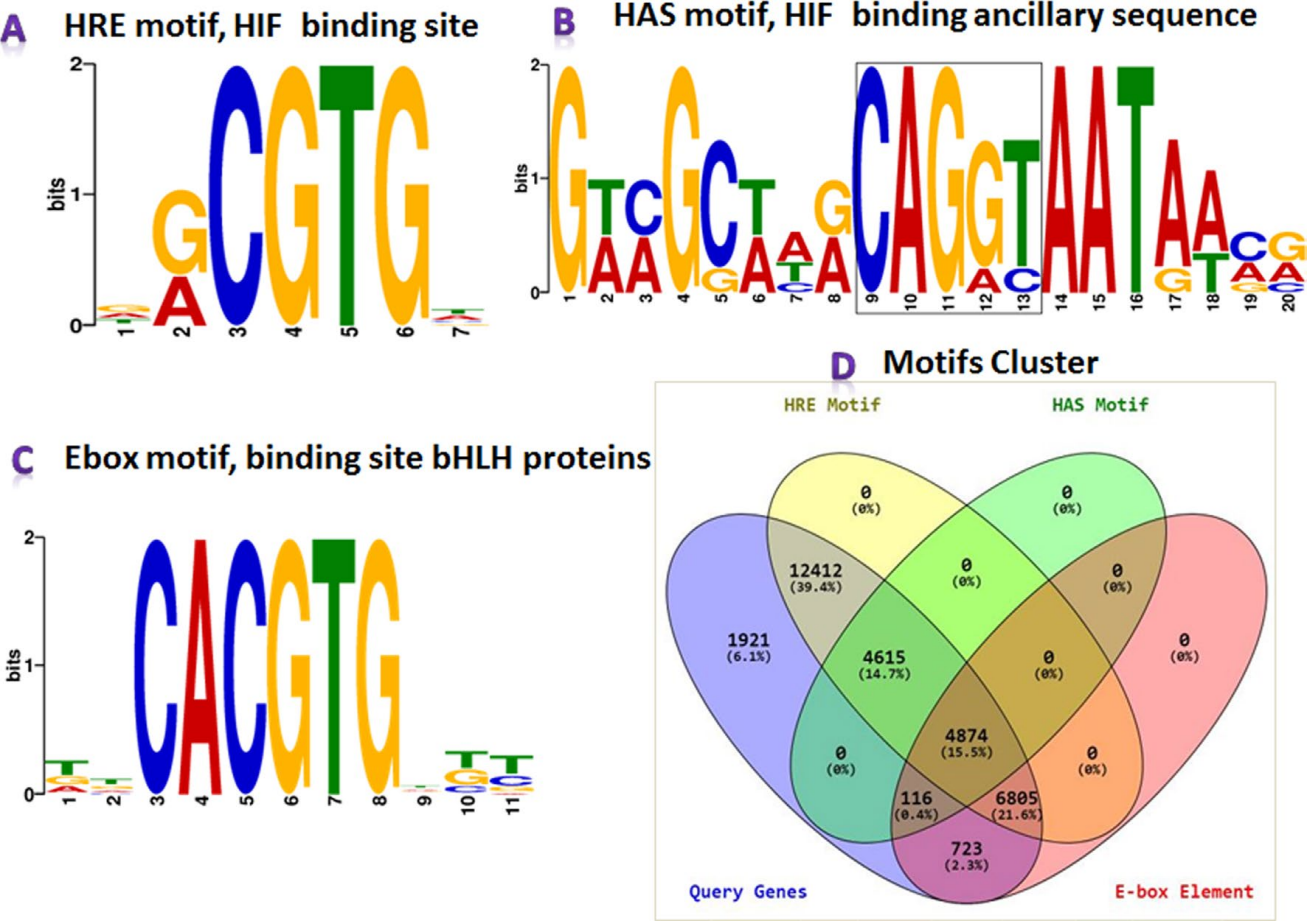
MEME-ChIP program categorized the obtained motifs into **(A)** HRE, **(B)** hypoxia ancillary sequence (HAS), and **(C)** E-box. **(D)** Clustered information on HRE, HRE with HAS, E-box, and E-box with HAS presented in the Venn diagram.

MEME-ChIP results revealed that the HREFinder identified motifs in the upstream sequence were true HRE with HAS motifs, but several HRE motifs were identified as E-box (5′-CACGTG-3′) elements, i.e., binding site of class bHLH TF. HIF also belongs to bHLH TF but does not bind with the E-box motif for inducing gene expression (Benita et al., 2009; Schödel et al., 2011). The results of the MEME analysis suite for motif validation is presented in **Figures 3A–C**. Different motifs in various combination were found in the analysis that included HRE, HRE with HAS, HRE with E-box, E-box, and E-box with HAS.

Again, a Perl program “MotifClust.pl” was designed for preparing a suitable dataset for clustering different motifs with their cumulative frequency distribution for presenting them in a Venn diagram (**Figure 3D**). The program generated four datasets: i) all genes taken under this study, ii) all genes containing HRE, iii) all genes containing HAS, and iv) all genes containing E-box. These datasets were submitted into Venny tool, and a Venn diagram, presenting clusters of different motifs, was generated. In the analysis, upstream of 28,706 genes containing HRE motifs were identified, where 4,615 genes in upstream had HRE with HAS. Additionally, the E-box was obtained in the upstream sequences of 12,518 genes, in which 11,679 sequences contained both HRE and E-box motifs.

### HREExplorer: An Information Browser and Data Analyzing Facility

HREExplorer is a web-based platform developed using LAMPP and JavaScript for displaying information obtained on HRE mining and meta-analysis in the genome of *C. carpio*. This data delivering system has the ability to display comparative analysis of findings with previously identified hypoxia-responsive genes of zebrafish and humans.

**Figure 4:**
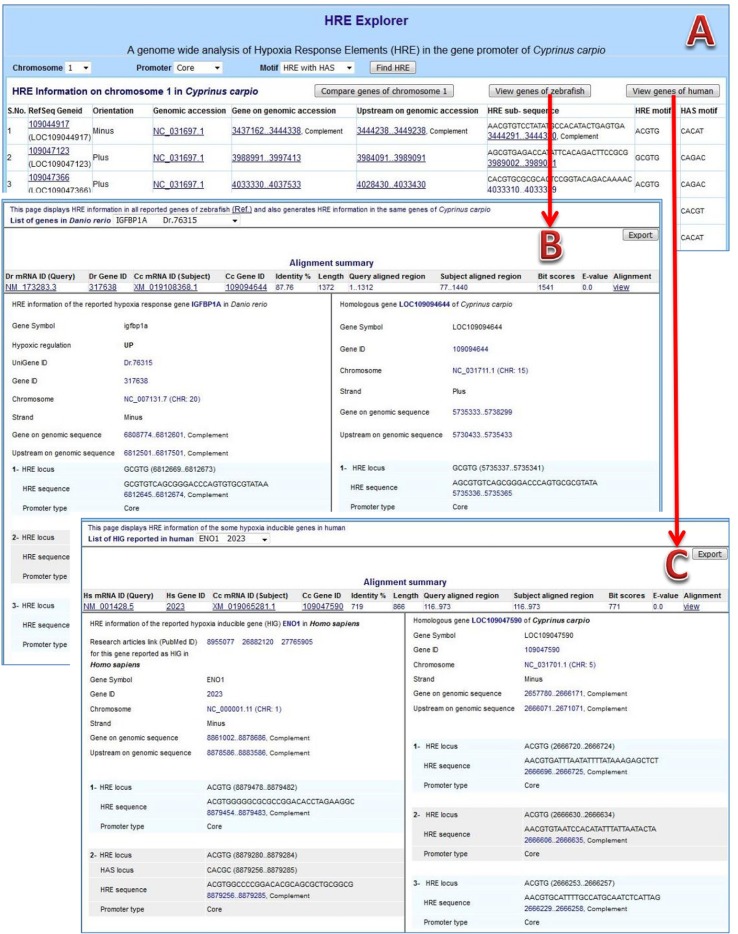
HREExplorer. **(A)** Genome-wide analysis of HRE in the promoter region of *C. carpio*. **(B)** Screenshot showing a comparative view of possible HIF binding sites (HBS) in the promoter of homologous gene in zebrafish and *C. carpio*. **(C)** Screenshot showing comparative view of possible HBS in the promoter of the homologous gene in humans and *C. carpio*.

**HRE information browser:** The content of the prepared GFF file was managed into tables of the database for browsing the information on Web. The complete result of HRE mining and their analytical views are available at URL: http://mail.nbfgr.res.in/HREExplorer. The homepage of HREExplorer provided a selection list of all *C. carpio* chromosomes as well as user-friendly browsing facilities for visualizing HRE and HRE with HAS motifs in particular promoter region within a selected chromosome (**Figure 4A**). The displayed information is hyperlink and leads to the respective pages of the NCBI database for cross-validation of the information and viewing the sequences of a particular region.

### Comparative Analysis With Hypoxia-Responsive Genes of Zebrafish

The 5 kb upstream sequences of 737 differentially expressed genes of zebrafish were used for mining HRE and HRE with HAS. The HRE sites were obtained in 679 genes with high frequencies in core promoters, especially near the TSS of genes, in zebrafish. There were 270 up-regulated genes among those 679 genes and 91 genes among them were HRE with HAS. A total of 298 HREs with HAS elements within 30 nt stretch in downstream region were obtained in 237 distinct genes. Further, a comparative analysis between the orthologous genes of zebrafish and *C. carpio* was done. HREExplorer presents a button “*View genes of zebrafish*” for generating and exporting comparative analysis information on RHRG of zebrafish along with homologous genes of *C. carpio*. This page displays a selection list of RHRG in zebrafish and displayed information on selected gene along with same gene information on *C. carpio*, which includes homology search summary, sequence alignment, information on chromosome, localization strand, gene upstream and HRE and HAS motifs with promoter location (**Figure 4B**).

The homepage also displays a button “*Compare genes of chromosome*” which leads on another page displaying matching gene information on *C. carpio* with HRG in zebrafish on homology based. This page is different form “*View genes of zebrafish*” because it provides information on homologous genes to zebrafish on a selected chromosome of *C. carpio*. A similarity search was also performed in the comparative analysis, where the conserved sequence in an orthologous gene of both species might be validated as a bonafide HRE binding site in the gene of *C. carpio*.

### Comparative Analysis With HIF Targeted Genes of Humans

The upstream sequences of human HIG genes were parsed from human genome (Assembly: GCF_000001405.38) and HRE sites in each upstream sequence were mined. The information about genes, PubMed ID of relevant article in which those genes reported as HIG in humans and localization of upstream along with total number of HRE in different promoters were reported, which are presented in **Table 2**. This information were used for performing comparative analysis between homologous genes of humans and *C. carpio*. HREExplorer also facilitates the user for comparative analysis between the HRE localized regions in *C. carpio* and the HRE containing region of the orthologous gene reported for hypoxia response in humans. The homepage displays a button “*Comparative analysis of human HIG*,” which leads to another page displaying matching gene information on *C. carpio* with HIG in humans on homology. This page presents a selection list of HIGs in humans and displays information on selected genes along with same gene information on *C. carpio*, which includes homology search summary, sequence alignment, and PubMed ID of the research article in which these genes reported as HIG in humans. Information on chromosome, localization strand, gene upstream, HRE, and HAS motifs with promoter location are displayed (**Figure 4C**).

**Table 2:** List of hypoxia-inducible genes (HIGs) reported in humans.

S.N.	PubMed ID	Gene ID (symbol)	Chromosome (strand)	Upstream location	Total HRE sites
1.	8955077, 28610954, 27793029	226 (ALDOA)	16 (+)	30048190..30053190	8
2.	10777486, 15741220, 15031665	1356 (CP)	3 (−)	149221945..149226945	5
3.	11278891, 10594042, 15615775	1387 (CREBBP)	16 (−)	3880627..3885627	9
4.	23530034, 28478800, 18292194	1490 (CCN2)	6 (−)	131951278..131956278	1
5.	11278891	1906 (EDN1)	6 (+)	12251563..12256563	4
6.	8955077, 26882120, 27765905	2023 (ENO1)	1 (−)	8878586..8883586	11
7.	30781443, 17002676, 12239177	2056 (EPO)	7 (+)	100715900..100720900	4
8.	21382012, 24610033, 22866201	2321 (FLT1)	13 (−)	28495028..28500028	8
9.	27765905	3099 (HK2)	2 (+)	74827755..74832755	1
10.	17887916, 27582105, 10814519	3162 (HMOX1)	22 (+)	35376167..35381167	4
11.	21224490	3240 (HP)	16 (+)	72049692..72054692	3
12.	23844025, 7499259	3263 (HPX)	11 (−)	6440924..6445924	2
13.	17509524, 27560636, 27487118	3479 (IGF1)	12 (−)	102481739..102486739	3
14.	23363253	3638 (INSIG1)	7 (+)	155292872..155297872	11
15.	26269128, 26212717, 28193910	3939 (LDHA)	11 (+)	18389489..18394489	4
16.	8955077, 23437403, 25807933	3945 (LDHB)	12 (−)	21657871..21662871	2
17.	30547064, 25684657, 21450070	3952 (LEP)	7 (+)	128236301..128241301	7
18.	19307731, 21930697, 24026678	4151 (MB)	22 (−)	35623254..35628254	5
19.	18206644	4504 (MT3)	16 (+)	56584437..56589437	5
20.	27765905	5230 (PGK1)	X (+)	78099269..78104269	8
21.	23363253	6236 (RRAD)	16 (−)	66925436..66930436	5
22.	17442736, 15525582, 10401038	6513 (SLC2A1)	1 (−)	42959076..42964076	4
23.	9242677, 26183475	7018 (TF)	3 (+)	133657086..133662086	4
24.	10446188	7037 (TFRC)	3 (−)	196082061..196087061	7
25.	11056166, 10607702, 30781443	7422 (VEGFA)	6 (+)	43765309..43770309	5
26.	24037094, 19920186, 23363253	8660 (IRS2)	13 (−)	109786467..109791467	2
27.	23363253	10458 (BAIAP2)	17 (+)	81030231..81035231	2
28.	27765905, 28760743	29923 (HILPDA)	7 (+)	128450930..128455930	9
29.	27765905	51537 (MTFP1)	22 (+)	30420723..30425723	7
30.	27765905	79001 (VKORC1)	16 (−)	31094899..31099899	5

### GitHub Resource

The present study incorporated a set of in-house Perl program (**Table 1**), which might be used either to add new species in this comparative analysis with *C. carpio* or to perform genome-wide comparative analysis between two species of the same family. Those in-house programs have been uploaded to GitHub (https://github.com/iliyasrashid/GWHREAnalysis) along with “readme” and publicly available for researchers.

## Discussion

There are several reports on the *in silico* identification and prediction of HBS in the genome-wide analysis of mammals and fish (Benita et al., 2009; Ortiz-Barahona et al., 2010; Schödel et al., 2011; Greenald et al., 2015). Adapting those approaches, *C. carpio* was selected for the genome-wide mining and analysis of HIF-1 binding sites due to its tolerability against hypoxia, availability of complete genome sequence, and wide distribution of the species. This type of studies provides many functional binding sites in the promoter regions of the protein coding genes, which might also be helpful in the comparative gene analysis with hypoxia-sensitive species.

The core promoter element, located in the upstream region of the gene adjacent to TSS and forming a platform for transcription initiation, is essential to initiate gene expression (Juven-Gershon and Kadonaga, 2010). High (>40%) frequency of HBS was observed in the core promoter region of the genes responsible to hypoxia (Schödel et al., 2011). However, HBS was not found in the core promoter in a well-recognized HIG EPO due to its distant location from TSS in the promoter region (Benita et al., 2009). HBS was identified as 5 kb far upstream of TSS in a mammalian HIF-1 targeted gene “eNOS” (Coulet et al., 2003). Hence, it might be concluded that the core promoter region is enriched with HIF-1 binding site, but some HIF-1 targeted genes also contain HIF-1 binding site in their proximal and distal promoter regions. Fragments of 3 to 5 kb far upstream from TSS, including a few hundred bases of downstream, were used for the analysis of the upstream region to detect the promoter and TFB of a gene (Ortiz-Barahona et al., 2010; Greenald et al., 2015). In this study, a set of 5 kb upstream sequences were taken for the mapping of hypoxia-binding sites in all protein coding genes of *C. carpio*. All mined HREs in those sequences were categorized by their frequency distribution in different regions, like core (0–1,000 nt), proximal (1,000–3,000 nt), and distal (3,000–5,000 nt) promoters. The method applied here for parsing 5 kb upstream sequences is not without limitations because there might be some genes whose intergenic region could be a few hundred or thousand but less than 5 kb. The methodology for parsing 5 kb upstream sequences uniformly of protein coding genes can cover a full or partial part of an adjacent gene located on its 5′ end.

In the promoter analysis of genes, the 500-nt consecutive fragments covering the entire length of the upstream were observed with almost uniform HRE distribution, but the higher frequency in the core promoter, especially near the TSS, justifies previous observation for HBS localization near the TSS (Schödel et al., 2011). In this, the *de novo* motif analysis, another motif, E-box, was observed along with HRE and HAS in the set of more or less 40-nt sequence fragment derived from the HRE location. An E-box consensus begins with a preceding C (5′-CACGTG-3′) in the HRE consensus (5-′ACGTG-3′) that serves as a TFB for the bHLH TF family. HIF-1 also belongs to the same transcription family, but it rarely binds with the E-box motif for inducing gene expression (Benita et al., 2009). There were 2.3% such genes identified from the total dataset, which contained only E-box, and were not used for further analysis.

*C. carpio* genes containing HRE were used in the comparative analysis with differentially expressed genes under hypoxia in zebrafish reported in the Supplementary file 1 of Greenald et al. (2015). The comparative study was performed for the further validation of our findings because <1% of the total HRE distributed in genomes is functionally active HRE only (Schödel et al., 2011); additionally, HIF also induces several genes having no HRE or any distinct HBS (Benita et al., 2009). In this way, a comparative study with experimental dataset provides a significant potential to validate the findings of the study. Zebrafish was used here as a reference species for comparative analysis because i) it is a model fish and close relative to *C. carpio* in evolution (Betancur-R et al., 2013), ii) the evolutionary lineage of genes shares a common ancestor in both species (Pasquier et al., 2016), iii) it has a well-characterized genome information available publically (Ruzicka et al., 2015; Faber-Hammond et al., 2016), iv) a vast collection of reported hypoxia-responsive genes is available in zebrafish (Greenald et al., 2015), and v) the information on zebrafish is used in the comparative analysis of *C. carpio* genome with significant findings (Henkel et al., 2012). Thus, based on the high degree of similarity and compactness of genome, the reported genes of zebrafish were further analyzed for mining of HRE and HRE with HAS, Thereafter, a comparative analysis between the same genes of *C. carpio* and zebrafish was performed. The HRE mining work was carried out in the already published data of differentially expressed genes of zebrafish because there was no information about motif localization and HBS in the downloaded file. A comparative analysis of our result with the dataset of zebrafish is not possible without HRE information in the upstream region of those genes. The HRE mining and analysis in the dataset of zebrafish revealed that 58 genes, reported in the list without any HRE elements in their 5 kb upstream sequences, were mostly down-regulated. The lack of HIF-1 binding site in 5 kb upstream sequences clarified that the down-regulated genes have no HIF-1 binding site for inducing gene expression during hypoxia, which supports the results about those down-regulated genes reported in zebrafish under hypoxia. Many up-regulated genes without any HRE elements in their promoter regions were also reported in zebrafish. A gene without HRE elements in their upstream sequence up-regulated during hypoxia strongly indicates that there might be another kind of motif to serve as an HIF-1 binding site. A unique HIF-1 binding site was previously identified in the lactate dehydrogenase B gene in killifish (Rees et al., 2009). In the analysis of zebrafish data, HRE elements were obtained in many genes reported for down-regulation. Many genes were identified with an obvious HBS in their promoters but reported for down-regulation. This might be due to the involvement of repressor proteins that bind and inactivate HIF-1 TF. HIF-repressing protein was reported that interacts with HIF-1 and put it off for inducing gene expression during hypoxia (Mahon et al., 2001). Hypoxia-tolerant fishes reduce their metabolic and physiologic activities during hypoxia to maintain homeostasis for coping with the conditions (Nikinmaa and Rees, 2005).

Zebrafish is reported as a hypoxia-tolerant species where several genes undergo down-regulation during hypoxic condition, which provides strong evidence in favor of the tolerance potential of this species. Earlier reports revealed that on, an average, 70% HIGs did not contain any recognized HBS in their core, proximal, and distal promoters (Mole et al., 2009; Xia et al., 2009). The analysis of up-regulated genes without any detectable binding sites in their upstream region also supports and verifies previous reports. The reports on the mined HRE and predicted HBS in the upstream sequences of protein coding genes strongly indicate that all HBS are not functionally active, which need robust analyses. The set of HRE elements along with few bases long upstream and downstream flanking regions were used for the optimization of HRE elements on a threshold score (Benita et al., 2009). This approach was applied here for the comparative analysis to find conserved sequences across a set of sequence fragments containing HRE with flanking regions. The comparative analysis confirmed that the HRE region in the genes, as reported in zebrafish, was found conserved with the HRE region of the same genes of *C. carpio*.

The method presented here for the comparative study is not without limitation, as several well-recognized genes, responding to hypoxia (like EPO, IGFBP1, etc.), could not be obtained in comparative analysis. Only the list of differentially expressed genes reported in zebrafish was used for the comparative analysis. However, genes such as EPO were missing in the reported list and were not obtained in the results but were earlier reported in the same species (Paffett-Lugassy et al., 2007). The selection of the tissue types for the study of the differential profiling expression of a gene or set of genes also plays a big role. Highly induced expression of EPO genes under hypoxia was previously reported in kidney tissue (Westenfelder et al., 1999). Moreover, few hypoxia-responsive genes, including Phd3, contain a hypoxia-binding site very far, about 12 kb, from the TSS toward upstream (Pescador et al., 2005). Some genes that contain a hypoxia-binding site far away from the TSS will not be present in results due to constraints in methodology of parsing only 5 kb upstream sequences. If the size of parsing the upstream is increased, then several genes are screened out because *C. carpio* genome contains many such genes whose upstream sequences (intergenic regions) are not >5 kb. Further, extensive analysis of the already reported genes for down-regulation in zebrafish during hypoxia revealed that either having HRE motifs apart from core region or lacking HAS element in the core promoter might be a clear evidence of down-regulation of those genes. However, HBS of 30 HIG in humans was also used additionally for comparative analysis with *C. carpio*, but an mRNA-based homology search did not show significant Blast hits due to distant relation between the two vertebrates (Roest Crollius and Weissenbach, 2005). The mRNA sequences of only 20 of 30 genes of human HIG showed hits with mRNA sequences of *C. carpio* and considered those for the comparative analysis in this study.

An information browser, HREExplorer, was developed for viewing information on genes with analytical ability to facilitate a comparative view of the genes’ promoter of *C. carpio* along with promoters of hypoxia-responsive genes of zebrafish and humans. HREExplorer enriched with an interactive user interface that displays information such as chromosome and a specific fragment-like gene, upstream and HRE regions, is hyperlinked to the primary source of data for cross-validation of information and sequences.

## Conclusion

A genome-wide analysis of hypoxia-binding sites in protein coding genes in the hypoxia-tolerant species *C. carpio* was performed. All mined HRE and HRE with HAS were analyzed with Position Weight Matrices (PMW-based) tool for motif validation. Further, the results were compared with differentially expressed genes under hypoxia in zebrafish and reported HIG of humans for the identification of bonafide HBS. Additionally, the localization of possible HBS in different core, proximal and distal promoter regions was also explored in the comparative analysis. The results of analysis are available on the web server that can be seen using HREExplorer for each chromosome. The web browser also facilitates a comparative analysis of HRE regions between orthologous genes of *C. carpio* versus zebrafish and *C. carpio* versus human. The outcome of the genome-wide analysis and HRE identification of the possible HBS in 5 kb upstream sequences of protein coding genes in *C. carpio* are useful for performing comparative analysis and validating the findings in other fishes. More hypoxia-responsive genes reported in zebrafish and human might be included in the future for comparative analysis and validating HBS in more genes of *C. carpio*. The information generated in undertaken species will help in evaluation of HIF targeted genes in the close relative species of the carp family, especially in Indian major carps. The functionality of the randomly distributed HRE site in the genome is not much explored in previous studies, but their distribution in the core and proximal promoters along with HAS might be functional sites in hypoxic gene regulation. The potential discovery of canonical HRE demonstrated under the context of genome-wide analysis would provide a foundation toward a better analysis for identifying hypoxia-responsive elements and their active involvement in hypoxia regulation.

## Author Contributions

IR, RK, and PS conceived the study and the conceptual design of the work. IR collected and compiled the data programmed for data mining and integration, designed the interface, and developed the database and application modules for browsing and analyzing the data. AKP tested the workflow model and application modules. RK, BK, MS, and MS supported for fish biology information. IR, AKP, and PS drafted the manuscript. All authors have read and approved the manuscript.

## Conflict of Interest Statement

The authors declare that the research was conducted in the absence of any commercial or financial relationships that could be construed as a potential conflict of interest.
